# Emergence of ST63 Pandrug-Resistant *Acinetobacter pittii* Isolated From an AECOPD Patient in China

**DOI:** 10.3389/fcimb.2021.739211

**Published:** 2021-10-14

**Authors:** Ling Yang, Ning Dong, Chen Xu, Lianwei Ye, Sheng Chen

**Affiliations:** ^1^ Department of Laboratory Medicine, The First Affiliated Hospital of Guangzhou Medical University, Guangzhou, China; ^2^ Department of Infectious Diseases and Public Health, Jockey Club College of Veterinary Medicine and Life Sciences, City University of Hong Kong, Hong Kong, Hong Kong, SAR, China; ^3^ State Key Lab of Chemical Science and Drug Discovery, Department of Applied Biology and Chemical Technology, The Hong Kong Polytechnic University, Hong Kong, Hong Kong, SAR, China

**Keywords:** pandrug-resistance, *Acinetobacter pittii*, AECOPD, whole genome sequencing, *bla*_OXA-58_, point mutations

## Abstract

*Acinetobacter* sp. is among the ESKAPE organisms which represent the major nosocomial pathogens that exhibited a high resistance rate. *A. pittii*, frequently associated with antimicrobial resistance particularly to carbapenems, is one of the most common *Acinetobacter* species causing invasive infection. Pandrug resistant *A. pittii* has rarely been reported. Here, we report the case of a patient with acute exacerbations of chronic obstructive pulmonary disease three years after double lung transplantation and developed severe pneumonia associated with pandrug resistant *A. pittii* infection. Phenotypic and genomic characteristics of this pandrug resistant isolate (17-84) was identified, and the mechanisms underlying its resistance phenotypes were analyzed. Isolate 17-84 belonged to ST63, carried a non-typable and non-transferable plasmid encoding multiple acquired resistance genes including carbapenemase gene *bla*_OXA-58_. Point mutations and acquired resistance genes were identified which were associated with different drug resistance phenotypes. To our knowledge, this is the first detailed phenotypic and genomic characterization of PDR *A. pittii* causing severe infections in clinical settings. Findings from us and others indicate that *A. pittii* could serve as a reservoir for carbapenem determinants. The emergence of such a superbug could pose a serious threat to public health. Further surveillance of PDR *A. pittii* strains and implementation of stricter control measures are needed to prevent this emerging pathogen from further disseminating in hospital settings and the community.

## Introduction

*Acinetobacter* species are among the high-priority nosocomial pathogens which could cause severe infections among immunocompromised patients (Howard et al., 2012). Infections caused by *Acinetobacter* spp. could be untreatable as they exhibit the potential to develop resistance to a wide range of antibiotics, particularly carbapenems (Wong et al., 2019). Carbapenem resistance in *Acinetobacter* spp. is mainly caused by the production of carbapenem-hydrolyzing class D β-lactamases (CHDLs) including OXA-23-like, OXA-58-like, and OXA-24-like enzymes (Ayibieke et al., 2020). *Acinetobacter pittii*, formerly termed genomospecies 3 of the *A. calcoaceticus*-*A. baumannii* complex, is a close relative of *A. baumanii* which is increasingly recognized as a significant cause of hospital-acquired infections (Chusri et al., 2014). A recent multicenter investigation in Japan suggested *A. pittii* was the most common species causing invasive *Acinetobacter* infection (Kiyasu et al., 2020). The terms multidrug resistance (MDR), extensive drug resistance (XDR) and pandrug resistance (PDR) were used worldwide to define the non-susceptibility level of isolates, which are resistant to at least one agent in three or more antimicrobial categories, to at least one agent in all but two or fewer categories and to all agents in all categories, respectively (Magiorakos et al., 2012). MDR *A. pittii* including those resistant to antibiotics quinolones, carbapenems, etc., was reported across continents, yet PDR *A. pittii* was rarely reported (Gu et al., 2015; Brasiliense et al., 2019; Chen et al., 2019a; Cosgaya et al., 2019; Chopjitt et al., 2021). Here, we report the case of a patient with acute exacerbations of chronic obstructive pulmonary disease (AECOPD) three years after double lung transplantation (DLT) and developed severe pneumonia associated with PDR *A. pittii* infection. Currently available therapeutic options for such isolates remained limited and relied largely on antibiotics recently approved by the Food and Drug Administration (FDA) and European Medicines Agency (EMA) such as eravacycline, rifabutin and cefiderocol (Livermore et al., 2016; Trecarichi et al., 2019). This severe situation called for urgent action to prevent the healthcare-associated transmission of such PDR *A. pittii*.

## Materials and Methods

### Patient Information and Strain Isolation

In 2017, a 59-year-old male patient was admitted to hospital (Day 0) with a diagnosis of AECOPD. The patient underwent DLT three years before hospitalization and regular medical examinations revealed no abnormalities after DLT. On Day 2, the patient was referred to an intensive care unit (ICU) due to the development of severe symptoms complicated with type 2 respiratory failure. A CT scan showed inflammations and fibrosis, reduced transparency and patchy shadows in both lungs, inferring occurrence of bilaterally pleural effusion. He received mechanical ventilation and other supporting treatments. On Day 20, *Stenotrophomonas maltophilia*, *Acinetobacter* sp. and *Aspergillus* sp. strains were recoverable from the sputum sample. On Day 25, a sputum culture became positive for carbapenem-resistant *Acinetobacter* sp. Amphotericin B, imipenem, meropenem and polymyxin B were introduced. A tracheal aspiration culture on Day 34 was positive for carbapenem- and colistin-resistant *Acinetobacter* sp. From Day 35, lung infection worsened with giant pulmonary bulla and gradual decline in leukocytes is continued. The patient discharged following critical illness and was transferred to another medical center on Day 41. A sputum culture performed on Day 35 flagged positive for carbapenem- and colistin-resistant *Acinetobacter* sp. (isolate 17-84).

### Species Identification, Antimicrobial Susceptibility Testing, and Conjugation Assays

The species of the isolate was identified using the matrix-assisted laser desorption ionization/time of flight mass spectrometry (MALDI-TOF MS) (Bruker Daltonik GmbH, Bremen, Germany) and verified with the whole genome sequencing results. Antimicrobial susceptibility of 15 commonly used antibiotics (amikacin, gentamicin, kanamycin, meropenem, imipenem, ertapenem, cefotaxime, ampicillin, aztreonam, ciprofloxacin, colistin, tetracycline, tigecycline, trimethoprim-sulfamethoxazole, azithromycin) was tested using broth dilution method and interpreted according to the Clinical Laboratory Standards Institute (CLSI) guidelines, with the exception of tigecycline and colistin (CLSI, 2020). The breakpoint of colistin was interpreted according to the European Committee on Antimicrobial Susceptibility Testing (EUCAST) recommendations (EUCAST, 2020). No interpretive criteria are available for tigecycline from CLSI, EUCAST or FDA. Plasmid transferability was tested by conjugation using a rifampicin-resistant mutant of *A. baumannii* ATCC 17978 as the recipient strain. Presumptive transconjugants were selected using Mueller-Hinton II agar plates supplemented with meropenem (0.5 mg/L) and rifampicin (600 mg/L).

### Whole Genome Sequencing and Bioinformatics Analysis

Genomic DNA was extracted from overnight cultures by using the PureLink Genomic DNA Mini Kit (Invitrogen, Carlsbad, CA, USA). Whole genome sequencing was performed using both the Illumina HiSeq (Illumina, San Diego, CA) and the Oxford nanopore MinION (Oxford, UK) platforms. Hybrid assembly of both sequencing reads was conducted using Unicycler (v 0.4.4) (Wick et al., 2017). Complete genome sequence was annotated by the RAST tool and edited manually (Overbeek et al., 2013). Multilocus sequence typing (MLST) was conducted using MLST v2.1 (Seemann, 2021). Acquired antibiotic resistance genes were identified by ResFinder 4.1 (Bortolaia et al., 2020). Plasmid replicons were analyzed using the *A. baumannii* PCR-based replicon typing (AB-PBRT) scheme (Bertini et al., 2010). Insertion sequences (ISs) are identified using ISfinder (Siguier et al., 2006). Plasmid map was plotted using DNAPlotter v1.11 (Carver et al., 2009). Mutations in the *pmrCAB* and *lpxACD* operons which were associated with polymyxin resistance were determined by aligning their amino acid sequences of strain 17-84 with that of a polymyxin-susceptible strain *A. pittii* ST220 (NZ_CP029610) (Zhang et al., 2018). Point mutations conferring resistance to quinolones (*gyrA*/*B* and *parC*/*E*) were detected by comparing the coding sequences in the 17-84 genome with the corresponding sequences in the genome of an ST63 quinolone-susceptible *A. pittii* strain in our collection (data not shown).

## Results and Discussion

Isolate 17-84 was identified as *A. pittii*, displaying a PDR profile to aminoglycoside (amikacin, gentamicin, kanamycin), β-lactam (meropenem, imipenem, ertapenem, cefotaxime, ampicillin, aztreonam), quinolone (ciprofloxacin), polymyxin (colistin), tetracycline (tetracycline), sulfonamide (trimethoprim-sulfamethoxazole) and macrolide (azithromycin). MIC values and the associated mechanism of resistance are shown on **Table 1**.

**Table 1:** Results of antimicrobial susceptibility tests and genetic characterization.

Antimicrobial agents	MIC (mg/L)	Interpretation ^a^	Resistance genes	Mutations
**Aminoglycoside**				
amikacin	>128	R	*aph(3’)-VIb*	—
gentamicin	>128	R	*aac(3)-IId*	
kanamycin	>128	R		
**β-lactam^b^**				
meropenem	>16	R	*bla*_OXA-58_	—
imipenem	>16	R	*bla*_PER-1_	
ertapenem	>16	R		
cefotaxime	>16	R		
ampicillin	>128	R		
aztreonam	>128	R		
**Quinolone**				
ciprofloxacin	>16	R	—	*gyrA* (S81L); *gyrB*(A414T); *parC* (S84L)
**Polymyxins**				
colistin	4	R	—	*pmrC* (Q143L, L146F, Q148K, I171V, L258S)
**Tetracycline**				
tetracycline	>64	R	RND family efflux pump genes *adeDE*, *adeN*-*adeIJK*, *adeL-adeFGH*, *adeRS-adeAB*	
**Glycylcycline**				
tigecycline	2	NA		
**Sulfonamide**				
trimethoprim-sulfamethoxazole	>76/4	R	*sul2*	—
**MLS - Macrolide, Lincosamide and Streptogramin B**				
azithromycin	>64	R	*msr(E)*; *mph(E)*	—

MIC values are categorized as susceptible, intermediate, or resistant following CLSI document M100-S30 or the EUCAST breakpoints. Antimicrobial resistance genes and mutations conferring resistance phenotypes are presented.

^a^R, resistant; NA, no interpretive criteria are available for Acinetobacter spp. from CLSI, EUCAST or FDA.

^b^bla_ADC-25_ and bla_OXA-51_ genes were also detected on the chromosome of the genome, but not in association with an IS.

Isolate 17-84 contained a 3,914,011 bp chromosome and a 108,715 bp plasmid designated as p17-84_OXA. It belonged to ST63 *A. pittii* (Pasteur Scheme). Resistance genes including *bla*_ADC-25_ and *bla*_OXA-51_ both not associated with insertion sequences were identified on the chromosome of isolate 17-84. *bla*_ADC-25_ and *bla*_OXA-51_-like genes were both naturally occurring β-lactamase-encoding genes in *Acinetobacter* spp. which does not contribute to intrinsic β-lactam resistance unless an insertion sequence such as IS*Aba1* is located upstream (Zhou et al., 2020). Previous studies have reported the mutations on different chromosome-encoded genes associated with quinolone, polymyxin and tetracycline resistance among *Acinetobacter* spp. In isolate 17-84, quinolone resistance-associated mutations in topoisomerase *parC* (S84L), DNA gyrase *gyrA* (S81L) and *gyrB* (A414T) genes were detected. Among these, *gyrA* (S81L) and parC (S84L) target site mutations have been demonstrated to lower the affinity for quinolone, while the function of A414T mutation in *gyrB* remained to be investigated (Hujer et al., 2009). The major mechanisms of resistance to colistin in *Acinetobacter* sp. included complete loss of lipopolysaccharide (LPS) resulting from mutations in *lpxACD* genes, and phosphoethanolamine addition to LPS mediated through mutations in the *pmrCAB* operon (Beceiro et al., 2014). Mutations in the *pmrC* gene including Q143L, L146F, Q148K, I171V, and L258S were detected in strain 17-84 and whether these mutations contributed to the colistin resistance phenotype remined to be studied. No acquired tetracycline resistance determinant was observed in strain 17-84, and its resistance to tetracycline could be associated with the presence of diverse efflux pump genes (*adeDE*, *adeN*-*adeIJK*, *adeL-adeFGH*, *adeRS-adeAB*) belonging to the resistance-nodulation-division (RND) family that were reported to confer multidrug resistance (Pagdepanichkit et al., 2016). The function of these efflux pump genes warrant further verification.

**Figure 1:**
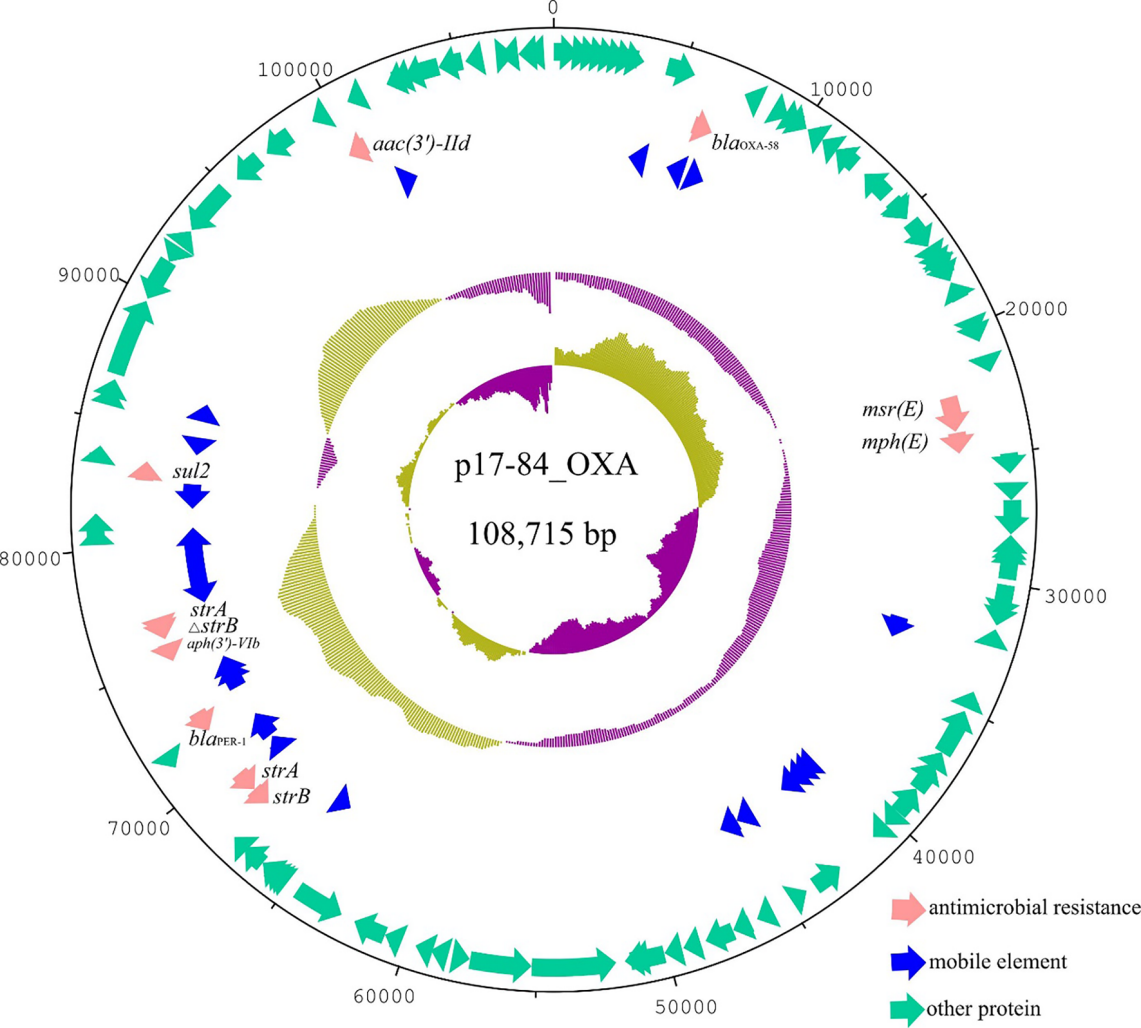
Circular plasmid map of p17-84_OXA. Pink, blue, and green arrows indicated the antimicrobial resistance genes, mobile elements and other ORFs, respectively. Antimicrobial resistance genes carried by the plasmid were labelled.

Plasmid p17-84_OXA contained 132 ORFs with a GC content of 39.6%. It was 100% identical to the 105,591 bp plasmid pOXA58_100004 (CP027249) from an *A. pittii* strain isolated from Sichuan province in China at 90% coverage. Compared with pOXA58_100004, plasmid p17-84_OXA has lost the *floR* resistance gene and gained a *~*9 Kb fragment encoding hypothetical proteins. p17-84_OXA and pOXA58_100004 both carried multiple antimicrobial resistance genes, including *aph(3’)-VIb*, *aac(3’)-IId*, *bla*_OXA-58_, *bla*_PER-1_, *sul2*, *msr(E)*, *mph(E)* and *strA* (2 copies) and *strB* genes (**Figure 1**). *aph(3’)-VIb* and *aac(3)-IId* confer aminoglycoside resistance by encoding *O*-phosphotransferase-type and *N*-acetyltransferase-type aminoglycoside-modifying enzymes, respectively. *bla*_OXA-58_ and *bla*_PER-1_ confer β-lactam resistance, with the former encoding carbapenem-hydrolysing class D β-lactamase oxacillinase OXA-58. *bla*_OXA-58_ was bracketed by insertion sequences IS*Aba3* with the genetic structure of IS*Aba3*-*bla*_OXA-58_-IS*Aba3*. IS*Aba3* drove the overexpression of *bla*_OXA-58_, which is associated with imipenem resistance (Nguyen et al., 2020). *sul2* confers resistance to sulfonamide. *msr(E)* and *mph(E)* are associated with macrolide resistance. *strA* and *strB* genes confer resistance to streptomycin. p17-84_OXA was non-typeable by AB-PBRT and non-transferable by conjugation under the experimental conditions.

Infections caused by MDR or PDR *A. baumannii* have been widely acknowledged, whereas non*-baumannii Acinetobacter* spp. infections are rarely reported in China (Yang et al., 2012; Zarrilli et al., 2013; Al Atrouni et al., 2016). The phenotypical species identification methods available to most diagnostic laboratories were not able to accurately differentiate the species belonging to the *A. baumannii* complex (Vijayakumar et al., 2019). The technological limitations in species discrimination have led to underestimation of non*-baumannii Acinetobacter* species in human infections (Pailhories et al., 2018). This could have led to the rare report of infections caused by PDR *A. pitti* previously, even though the pandrug-resistance phenotype in *A. baumannii* complex is common in some clinical settings (Karakonstantis et al., 2020a; Karakonstantis et al., 2020b). According to a recent study from a French hospital, *A. pittii* was isolated more frequently associated with bloodstream infections than *A. baumannii*, highlighting the importance of *A. pittii* in clinical settings (Pailhories et al., 2018). Chen et al. reported the co-occurrence of two carbapenemase genes, *bla*_OXA-58_ and *bla*_NDM-1_, on a single plasmid in *A. pittii* (Chen et al., 2019b). Yang et al. (Yang et al., 2012) reported an outbreak of ST63 carbapenem-resistant *A. pittii* which carried a 45-kb novel *bla*_NDM-1_-bearing plasmid in a Chinese ICU. This evidence suggested ST63 *A. pittii* was readily transmissible and has the capacity to acquire antimicrobial determinants and become competent, which could pose serious threat on public health. *In vivo* emergence of resistance to last resort antimicrobials including tigecycline and colistin was reported to be quite common in *Acinetobacter* spp (Karakonstantis, 2020). The fitness cost associated with acquired resistance and the virulence of PDR *Acinetobacter* sp. remained a major issue for medical treatment (Karakonstantis, 2020).Besides, differentiating *A. baumannii* complex infection from colonization remains difficult and further complicated particularly in polymicrobial infections such as the case in the current study (Karakonstantis and Kritsotakis, 2021). We acknowledge the limitation that the antimicrobial resistance profiles of strain 17-84 to recently approved antibiotics including eravacycline, cefiderocol and rifabutin, which are potential last resort treatment options, were not tested (Karakonstantis et al., 2020c; Luna et al., 2020). A further study on the treatment of infections caused by such PDR *Acinetobacter* sp. is underway to provide more insights into the therapeutic scheme.

In conclusion, we reported a case of severe pneumonia due to *A. pittii* infection in a patient with AECOPD three years after DLT. Phenotypic and genomic characterization indicated the *A. pittii* strain belonged to ST63, harbored a *bla*_OXA-58_-bearing MDR plasmid, carried resistance-associated point mutations and efflux pump genes, and exhibited a PDR phenotype. To our knowledge, this is the first detailed characterization of PDR *A. pittii* causing severe infections in clinical settings. Attentions should be paid to this emerging pathogen to prevent it from further disseminating in hospital settings and the community.

## Data Availability Statement

The datasets presented in this study can be found in online repositories. The names of the repository/repositories and accession number(s) can be found in the article/supplementary material. The complete genome sequence of Acinetobacter pittii strain 17-84 was deposited in GenBank with accession numbers CP059479 (chromosome) and CP059478 (plasmid p17-84_OXA) under BioProject accession PRJNA648287.

## Ethics Statement

The studies involving human participants were reviewed and approved by the Ethics Committee of the First Affiliated Hospital of Guangzhou Medical University. The patients/participants provided their written informed consent to participate in this study.

## Author Contributions

LYang and ND collected and characterized the strain and participated in manuscript writing. ND, CX, and LYe performed the whole genome sequencing, bioinformatics analysis and the conjugation assay. SC designed and supervised the study, interpreted the data, and wrote the manuscript. All authors contributed to the article and approved the submitted version.

## Funding

This study was supported by the Natural Science Foundation of Guangdong Province (2018A030310170) and the Collaborative Research Fund from the Research Grant Council of the Government of Hong Kong SAR (C5026‐16G).

## Conflict of Interest

The authors declare that the research was conducted in the absence of any commercial or financial relationships that could be construed as a potential conflict of interest.

## Publisher’s Note

All claims expressed in this article are solely those of the authors and do not necessarily represent those of their affiliated organizations, or those of the publisher, the editors and the reviewers. Any product that may be evaluated in this article, or claim that may be made by its manufacturer, is not guaranteed or endorsed by the publisher.
